# Biodegradable Hydrogel Beads Combined with Calcium Phosphate Bone Cement for Bone Repair: In Vitro and In Vivo Characterization

**DOI:** 10.3390/polym14030505

**Published:** 2022-01-27

**Authors:** Po-Sung Fu, Jen-Chyan Wang, Pei-Ling Lai, Shih-Ming Liu, Ya-Shun Chen, Wen-Cheng Chen, Chun-Cheng Hung

**Affiliations:** 1 School of Dentistry, College of Dental Medicine, Kaohsiung Medical University, Kaohsiung 807378, Taiwan; posung.elegant@msa.hinet.net (P.-S.F.); jechwz@kmu.edu.tw (J.-C.W.); 2 Department of Dentistry, Kaohsiung Municipal Ta-Tung Hospital, Kaohsiung 80145, Taiwan; 3 Division of Prosthodontics, Department of Dentistry, Kaohsiung Medical University Hospital, Kaohsiung 807378, Taiwan; casting0118@gmail.com; 4 Dental Medical Devices and Materials Research Center, College of Dental Medicine, Kaohsiung Medical University, Kaohsiung 807378, Taiwan; 5 Advanced Medical Devices and Composites Laboratory, Department of Fiber and Composite Materials, Feng Chia University, Taichung 40724, Taiwan; 0203home@gmail.com (S.-M.L.); yaschen@fcu.edu.tw (Y.-S.C.)

**Keywords:** hydrogel, biodegradable, calcium phosphate bone cement (CPC), in vivo, osseointegration

## Abstract

This study evaluated the in vitro characterizations of biodegradable hydrogel beads with calcium phosphate bone cement (CPC). Commercial fast-setting CPC and hydrogel beads were compared with 25%-volume hydrogel in CPC (C/0.25) in vivo. The histological behaviors and absorption rates of CPC only, hydrogel beads, and hydrogel/CPC composite were measured and compared at 4, 8, and 12 weeks. The results indicated that the C/0.25 composite can be molded and does not disintegrate when immersed in the solution, but this delays the phase transition of the CPC into the product in the early reaction process. The osteoprogenitor D1 cell affinity of the C/0.25 composite was equally competitive with that of the CPC-only. Adding hydrogel beads to CPC did not inhibit cell proliferation as well as differentiation of osteoprogenitor cells. In vivo histological evaluations did not indicate any significant difference in the CPC-only, hydrogel-only, and C/0.25 composite after 4 weeks of implantation; however, significantly less residue was observed in the C/0.25 composite relative to the CPC-only after 8 weeks. After 12 weeks of hydrogel beads implantation, the hydrogel degraded substantially, creating vacancies that were subsequently occupied by a large amount of soft tissue. New bone was formed in large quantities in the C/0.25; therefore, the C/0.25 composite is a promising option for a wide range of dental, craniofacial, and orthopedic applications.

## 1. Introduction

The initial stability of an implant (i.e., favorable primary stability) refers to the quality of osseointegration between the implant surface and the surrounding bone tissue [1,2]. If the marginal bone around an implant has high bone density and sufficient bone quantity, the increased stability of the implant can enhance its clinical success rate [3]. Therefore, active bone repair for osseointegration is particularly essential when obvious bone defects are required under complex clinical conditions, which include trauma, infection, tumor resection, bone reconstruction of large bone defects caused by bone abnormalities, and destruction of the regeneration process (including bone atrophy and osteoporosis caused by vascular necrosis) [4]. Among the investigated strategies, hybrid materials are a promising strategy for accelerating the overall regeneration process in bone defect regeneration. Due to the diversity and complementarity of the inherent characteristics of the different types of materials, hybrid materials containing organic matter, such as the use of extracellular matrix (ECM) proteins, decellularized ECM or hydrogels, and inorganic components, are emerging as a very efficient composite [5,6,7,8].

In dentistry, granular fillers are commonly used for clinical osteogenesis. These filling materials are mainly xenograft or synthetic bones composed of β-tricalcium phosphate (β-TCP) or β-TCP combined with hydroxyapatite (HAp). However, the absorption rates of β-TCP and HAp complex are low, and at least 6 months is required to complete implantation [9,10]. In addition, the granular shape of granular filling materials results in another disadvantage: they are fragile in clinical bone repair surgery and difficult to form into shapes that conform to bone defects [11]. To improve the shortcomings of granular fillers, paste-, mud-, or slurry-like filling materials of calcium phosphate bone cement (CPC) with higher strength and strong formability have been developed [12,13,14]. However, after the reaction, the main product phase of CPC is HAp, which still requires a long time for absorption, and the CPC residue is brittle, which is not conducive for implantation. Therefore, adjusting the absorption rate and porosity of paste-like bone filling material is key to the application of CPC in alveolar bone restoration.

To increase the absorption rate of CPC, in addition to changing its composition, increasing its porosity is an option [15]. Hydrogels are composed of many hydrophilic functional bonds in the main chain molecules, which are naturally present in the form of polymer networks (e.g., gelatin, alginate, chitosan, silk fibroin, and collagen, which can also be made synthetically) [16]. Hydrogel is a 3D network structure with an entangled structure of hydrophilic bonds that are formed during the preparation process that allows hydrogel to absorb a large amount of water [17]. Due to cross-linking, dehydration, and the entanglement of physical hydrogen bonds in chains in the hydrogel, it usually does not dissolve quickly and can therefore retain its shape until it is completely degraded [18]. Thus, hydrogels are widely used in biomedicine and tissue engineering to guide tissue regeneration [19,20,21]. 

A study revealed a strategy to encapsulate cells in alginate beads; as CPC setting is harmful to MC3T3-E1 osteoblasts, alginate is used to encapsulate and protect the cells in the CPC [22]. The main advantage of adding hydrogel beads to CPCs is the ability to absorb large amounts of blood to release platelet-derived growth factors after implantation, rather than adding expensive purified growth factors. By following the degradation of the hydrogel, pores can be formed in situ. These pores can cause blood vessels to grow inward, promote the transport of progenitor cells, and accelerate tissue healing.

Therefore, to improve the shortcoming of insufficient pores for tissue ingrowth, a compromise strategy is to composite biodegradable hydrogels, such as alginate, gelatin, alginate/chitosan, hyaluronic acid, and silk fibroin protein, as a filler in CPC matrix [22,23,24,25,26,27,28]. Studies have pointed out that the addition of hydrogel particles after implantation can be used as a carrier, which means that the hydrogel can protect the carrier factor from the hardening mechanism of CPC for a certain time [26,29]. In addition, the degraded hydrogel after clinical surgery can form pores in situ. These holes can enhance blood vessels to grow inward, promote the transport of progenitor cells, and accelerate tissue healing [25]. According to our previous study [29], CPC composite hydrogel beads show controlled release of antibiotics, and the optimal content of hydrogel beads can be 25 vol.%, which has sufficient strength, antibacterial activity, and bio-reactivity. Continuing the previous research results, in this study, we selected 25 vol.% hydrogel beads to verify the in vitro and in vivo characteristics of the hydrogel beads/CPC composite. As the in vitro biochemical conditions are controlled and kept constant, the samples can be compared based on the difference in the loading of the hydrogel in CPC to obtain the optimal content. This study intended to use the minimum number of animals to study the in vivo bone filling model because it provides natural biological interactions between hydrogel/CPC composite and tissues to identify in vitro approaches that are sufficient to represent in vivo conditions.

In sum, the microstructure of hydrogel can absorb a large amount of blood during an operation, release platelet-derived growth factors from absorbed blood [30,31], and further induce blood vessel ingrowth when the space occupied by the hydrogel is degraded. The purpose of this study was to combine 2–3 mm spherical hydrogel beads with CPC for testing. The optimal conditions of the percentage of hydrogel bound to the hydrogel/CPC composite were determined per our preliminary research to prevent the dispersion of the hydrogel/CPC composites. We explored the physical and chemical properties of the developed composite and the cellular responses in co-cultured precursor osteoblasts on the composite in vitro. The effect of hydrogel/CPC composites in promoting bone repair was evaluated by observing the absorption rate at multiple implant time points in vivo.

## 2. Materials and Methods

### 2.1. Raw Materials

The raw materials that were used to make the hydrogel were gelatin (142062.1210, 80–100 Blooms (USP-NF, BP, Ph. Eur.) pure, pharmaceutical grade, PANREAC, EU; Pan Reac AppliChem, Darmstadt, Germany), sodium alginate salt of brown algae (sodium alginate; S1118, Spectrum Chemical, New Brunswick, NJ, USA), and N-(3-dimethylaminopropyl)-N’-ethylcarbodiimide hydrochloride (EDC, E-1769, molecular weight, 191.70 g/mol; Sigma-Aldrich, St. Louis, MO, USA). The fast-setting CPC used in the present study was developed in our previous research [14]; it is already commercialized, and the ratio of powder to hardening solution was implemented according to the manufacturer’s instructions (Realbone Technology Co., Kaohsiung, Taiwan).

### 2.2. Preparation of Hydrogel Beads

Porous spheres were fabricated through solvent casting and particulate leaching and the detailed procedures followed our previously described methods [29]. A colloidal suspension was prepared by mixing sodium alginate and gelatin at a *w/w* ratio of 1:4, heating to 50 °C, and suspending the mixture in 10 mL of deionized distilled (DD) water. Then, the suspension was mixed with saccharose particles in a particle:colloid ratio of 4 g:1 mL, and after the particles were leached, more than 70% of interconnected pores were formed in the gel. After the colloids were evenly mixed, we used an automatic injector to control the extrusive rate of colloids to produce macrospheres of a fixed volume. The fixed volume was added dropwise to a crosslinker solution of 1% EDC and 0.5% anhydrous calcium chloride at 4 °C. Subsequently, the gel macrospheres were removed and reimmersed in 1% EDC solution for 24 h to complete the crosslinking reaction. The crosslinked gel macrospheres were soaked in DD water for 3 h at 25 °C and were washed three times to leach the particles. The macrospheres were then dried in a vacuum. The air pressure was reduced to approximately 26 μbar through lyophilization for 3 d. The prepared hydrogel beads were 2–3 mm in size. All materials that were employed for subsequent in vitro and in vivo measurements (including hydrogel beads with 200 μm perforated pores, CPC powder, and hardening solution) were γ-ray-sterilized at 25 kGy (China Biotech Co., Taichung, Taiwan).

### 2.3. Preparation of Composite

The composite of CPC and 25%-volume hydrogel beads was prepared in situ for the experiment. To prepare the composite, the CPC powder and hardening solution were first premixed for 2 min (powder/liquid ratio = 2 g/mL) and the hydrogel beads were then added to the paste to form a composite slurry.

### 2.4. Characterization of CPC-Only and C/0.25 Composite

#### 2.4.1. Moldability and Disintegration Resistance

After the slurry of the C/0.25 composite was mixed, the C/0.25 composite was molded by hand to ensure that its plasticity allowed for it to be molded into the desired shape. After 5 min of mixing, the mold sample was placed in a container filled with water, and a photo was taken to observe the debris or sample collapse around the CPC-only and C/0.25 composite.

#### 2.4.2. Infrared Spectroscopy, Microstructure, and Phase Analysis

The functional group analysis of the materials used was conducted using an attenuated total reflection-Fourier-transform infrared spectrometer (FTIR, Nicolet 6700, Thermo Fisher Scientific, Waltham, MA, USA) and was analyzed for estimations. After the CPC-only was ground, the fine particles were uniformly distributed, so the transmission mode starting from 400 cm^−1^ could be adopted. However, as the hydrogel tended to agglomerate after grinding of the C/0.25 composite, the total reflection mode starting from 600 cm^−1^ was applied in the C/0.25 composite instead of the transmission mode.

Phase analysis of the CPC-only and C/0.25 composite was conducted through XRD and characterization. An X-ray diffractometer (XRD-6000, Shimadzu, Kyoto, Japan) with Ni-filtered Cu Kα radiation operated at 40 kV and 30 mA, and a scanning speed of 2°/min was used in the present study. Multiple phases of the composites were identified per the Joint Committee on Powder Diffraction Standards.

The fracture surfaces of the samples were examined using a field-emission scanning electron microscope (SEM; Hitachi S-3000N, Hitachi, Tokyo, Japan).

### 2.5. In Vitro Cell Culture Assays

#### 2.5.1. Relative Short-Term Morphological Observation of Cell Attachment

Bone marrow mesenchymal stem cells (MSCs) cloned from Balb/C mice (D1 cells) were purchased from the American Type Culture Collection (ATCC). The CPC and C/0.25 composite were converted into cylindrical samples with a diameter and height of 6 and 3 mm, respectively; they were then cultured in contact with D1 cells at a cell concentration of 1 × 10^4^ cells/mL in Dulbecco’s modified Eagle’s medium (DMEM) supplemented with 10% fetal bovine serum (FBS), 100 IU/mL penicillin, and 100 mg/mL streptomycin in an incubator at 37 °C in a humidified 5% CO_2_ atmosphere. The cell culture medium used was purchased from Gibco Thermo Fisher Scientific Inc., Waltham, MA, USA. A test piece was placed on a 48-well plate, and a culture medium was added to culture the cells. The cultivation time was 1 h, 1 day, and 2 days. After incubation was completed, the samples were washed sequentially with phosphate-buffered saline (PBS) and fixed with a mixture of 2.5% glutaraldehyde and paraformaldehyde. The specimens with cells were dehydrated with varying concentrations of alcohol, plated with metal, and then observed through SEM.

#### 2.5.2. Relative Long-Term Cell Proliferation, Mineralization, and Alkaline Phosphatase (ALP) Staining

After CPC was mixed with hydrogel beads for 2 min, the resulting C/0.25 composite was uniformly filled into a cylindrical stainless-steel mold (6 mm width and 3 mm depth) under a pressure of 0.7 MPa for testing. After 1 × 10^5^ D1 cells were inoculated on the surface of the specimen, a relatively long-term cell culture was conducted at intervals of 1, 4, 7, 10, and 14 days. DMEM supplemented with 10% FBS culture medium was changed 3 times a week. Cell metabolic activity and ALP production (an early marker of osteogenesis) were determined using an alamarBlue assay (Bio-Rad, Hercules, CA, USA) and p-nitrophenyl phosphate kits (pNPP; St. Louis, MO, USA). The assay is a nontoxic, cell-permeable compound that is blue in color and emits little fluorescence, and upon entry into the cell, resazurin is reduced to resorufin, a reddish, highly fluorescent compound. For comparison, the metabolic activity of the D1 cell control group was the group normalized by pure medium only at different culture intervals. That means the reduction of alamarBlue reagent as a correlate of cell metabolic activity and cell proliferation as evaluated via alamarBlue assay in the direct cell culture setting during the incubation period. After the culture process was completed, the cells on the sample surface were washed twice with PBS, transferred to a 900 μL medium containing 100 μL of alamarBlue reagent, and incubated for 4 h. An enzyme-linked immunosorbent assay plate reader (SPECTROstar Nano, BMG LABTECH, Offenburg, Germany) was used to measure the absorbance of the reaction medium with alamarBlue at 570 nm (595 nm was used as the reference wavelength). After the viability test was performed, the specimens were washed twice with PBS, transferred into individual wells that each contained 500 μL of pNPP substrate solution, and incubated for 30 min; the absorbance of the reaction solution was read at 405 nm. Each experiment was performed in triplicate, with replicates performed on different cell culture days (*n* = 3).

ALP staining was performed using the SIGMAFAST BCIP/NBT tablet (N2770, Sigma-Aldrich) as a substrate. The cells on the surface were fixed and washed with distilled water. The substrate solution was then added to the sample and incubated at 37 °C for 45 min. Before a general inspection was performed using an optical microscope, the ALP-stained test sample was washed thrice with distilled water.

### 2.6. Histological Observation In Vivo

The animal study performed in the present study was reviewed and approved by the Institutional Animal Care and Use Committee (IACUC) of Kaohsiung Medical University (IACUC 108004, 20 March 2019). The testing groups were the CPC, hydrogel beads, and C/0.25 composite groups. Twelve rabbits were randomly divided into three groups (corresponding to postoperation periods of 4, 8, and 12 weeks) and then sacrificed. The surgical procedure performed in the present study is identical to those of previous studies [32,33]. In the present study, male New Zealand White rabbits weighing 2.8–3.5 kg received implants. The New Zealand White rabbit is an animal model for the long-term biocompatibility testing of implant materials per American Society for Testing and Materials specifications and recommendations. After mixing the CPC uniformly and waiting 2 min, and the hydrogel beads were then mixed. As CPC has fast-setting properties to obtain sufficient strength, it will become a slurry-like paste with high viscosity. After composite mixing was performed for 1 min, the paste was loaded into a 3 mL needle-free syringe with the barrel roof left open and injected into the prepared bone cavity (4 mm [diameter] × 5 mm [depth]) in the distal malleolus (condyle) of the rabbit femur. The hydrogel spheres and C/0.25 composite were filled into the cavity by using surgical instruments. Retrograde injection and filling procedures were performed carefully from the bottom to the surface of the defect. When the rabbits were sacrificed, their femur portions were immediately excised, and excess tissue was removed. The sectioned bone was fixed, dehydrated, embedded in epoxy resin, and sectioned to a thickness of 250 μm. The samples were thinned out to a final thickness of 60 μm, polished, and glued to slides by using Permount (Fisher Scientific, Fair Lawn, NJ, USA).

The residual areas of the implantations were measured using an image analysis system. The total resorption bone area (%) was determined using the following equation:

Bone resorption ratio in % = (1 − [cross-sectional residual area of implant]/[cross-sectional area of original implant]) × 100%. (1)

The sections were stained with hematoxylin and eosin, and their histology was observed using optical microscopy (BX51, OLYMPUS, Tokyo, Japan).

### 2.7. Statistical Analysis

IBM SPSS 22 software (IBM Co., Armonk, NY, USA) was used to identify differences in cell proliferation, mineralization, and secretion capacity of ALP among the groups. Data from more than two groups were compared using two-way analysis of variance post hoc with the Tukey’s honestly significant difference (HSD). A probability (*p*) value of <0.05 was considered statistically significant.

## 3. Results and Discussion

### 3.1. Comparison of Physicochemical Properties of CPC-Only and C/0.25 Composite

#### 3.1.1. Moldability and Disintegration Resistance

**Figure 1:**
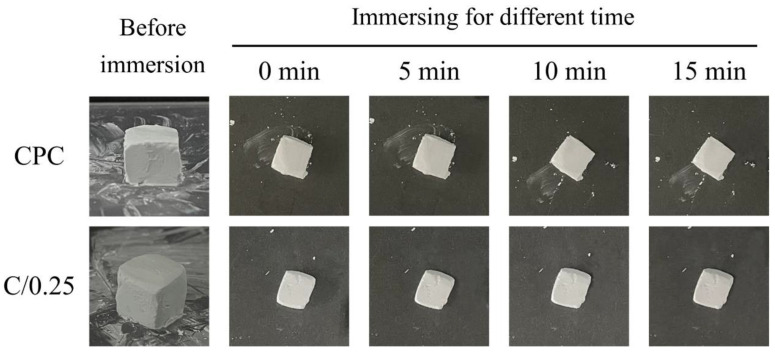
Comparison of moldability and disintegration resistance of fast-setting CPC-only and C/0.25 composite (cube size = 1 cm^3^).

A comparison of the operability of the CPC-only and C/0.25 composite revealed that after the CPC was mixed, it could be formed without a waiting period. For the C/0.25 composite, a paste was first uniformly mixed, after which 25%-volume hydrogel beads were added followed by 1 to 2 min of waiting. When the C/0.25 composite became clay-like, it resisted decomposition when placed in double-distilled water (ddH2O). Although a slight difference was observed in the operability of the CPC and C/0.25 composite, both groups allowed for easy shaping and did not disintegrate when an aqueous solution was introduced (Figure 1).

When bone cement is mixed with additives (e.g., composites that contain 5–12.5% gelatin particles), disintegration occurs [34]. The added hydrogel beads accounted for 25 vol.% of the C/0.25 composite, and no disintegration was observed, which may be related to the fast-setting CPC used in this study [35].

#### 3.1.2. Morphology of CPC-Only and C/0.25 Composite

**Figure 2:**
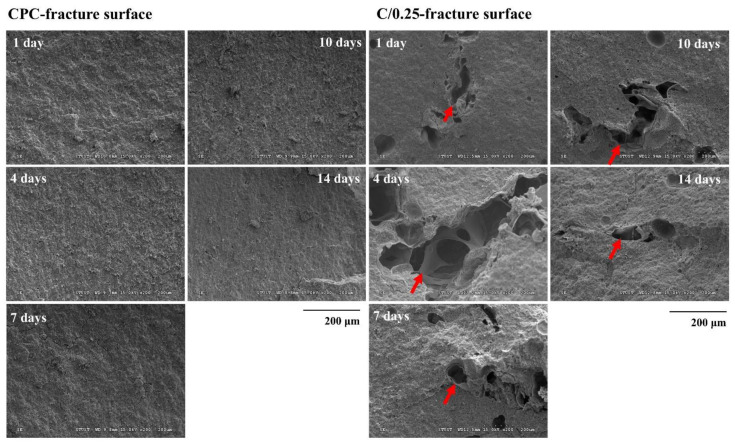
Microstructure analysis of fracture cross-section of CPC-only and C/0.25 composite at multiple immersion time points (arrow indicates hydrogel beads in CPC).

The uneven surface of the CPC-only fractures resembled coral reefs, and no obvious stomata were observed on the surface (Figure 2). When immersion time increased, pores began to appear, which could be caused by the gradual dissolution of the CPC after immersion. The porous structure of the hydrogel beads could still be observed at the fracture of the C/0.25, indicating that the perforated pores of the hydrogel beads were not blocked by the CPC paste mixture. An examination of the C/0.25 fractures revealed that the CPC matrix could completely cover the hydrogel beads, and the content of the hydrogel did not affect the adhesion and hardening of the CPC, such that the C/0.25 could maintain its high resistance to disintegration (Figure 1). However, the C/0.25 composite still exhibited several disadvantages. The hydrogel beads were added to the CPC and mixed uniformly to form a clay-like paste, which caused the composite to initially harden and, consequently, the composite paste to lose its injectability. If the repair part is not too complicated, it can also be molded by hand. For example, posterior lumbar fusion (PLF) or the need to heal large defects usually require bone grafts at some stage of the reconstruction process. The main consideration is the characteristics of the implant material itself rather than the injectability.

#### 3.1.3. Diffraction Patterns and Spectral Analysis

**Figure 3:**
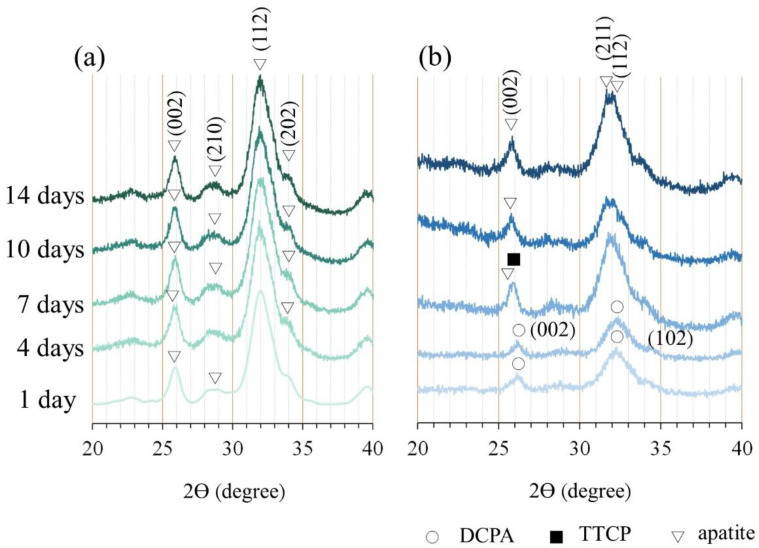
Diffraction patterns of (**a**) CPC-only and (**b**) C/0.25 composite. DCPA, dicalcium phosphate anhydrous; TTCP, tetracalcium phosphate.

After the CPC underwent an immersion reaction, its component phase transformed into a product phase that was mainly composed of hydroxyapatite (HA), and no diffraction peaks of the dicalcium phosphate anhydrous (DCPA) and tetracalcium phosphate (TTCP) formed after CPC-only immersion was observed (Figure 3a). The composition analysis of the C/0.25 composite at multiple immersion time points revealed that the diffraction plane of DCPA/(002) at 26.367° could be seen in the early stage of the reaction (Figure 3b). As the calcium cations in the alginate and gelatin-mixed hydrogel were combined with carboxyl groups, apatite crystals grew and precipitated in the hydrogel matrix through the preferential directional diffusion of phosphate anions. In particular, the planes of apatite diffractions corresponding to (211) and (112) increased, and the (210) plane of the apatite reduced [36]. When the immersion time increased, the diffraction peaks of DCPA disappeared and were replaced by the diffraction planes of apatite/(002) at 25.689° and apatite/(112) at 31.795° and 32.066°. After 7 days of immersion, the diffraction peaks of apatite gradually became dominant. The addition of hydrogel beads delayed the phase change of the CPC matrix; nevertheless, the final XRD phase of the C/0.25 composite was still converted into a product phase dominated by HA without the (210) plane after 14 days of immersion. The results showed that the interaction between the carboxylic acid of gelatin and the calcium cation of apatite can guide the growth of apatite with specific (211) and (112) planes (Figure 3).

**Figure 4:**
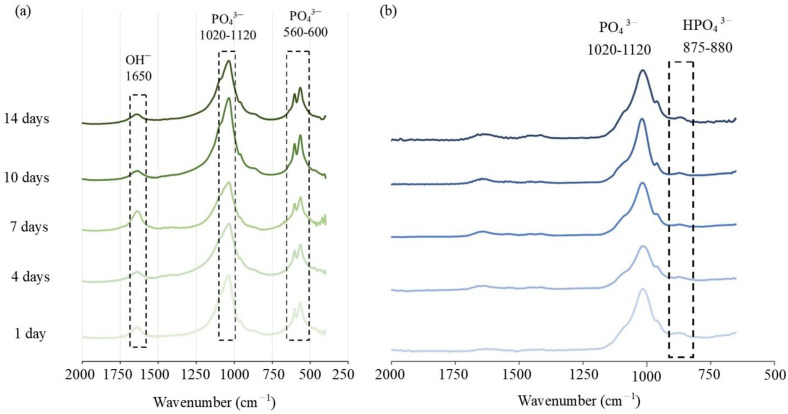
(**a**) CPC-only spectrum analysis adopting transmission infrared mode starting from 400 cm^−1^, and (**b**) the total reflection mode spectra of the C/0.25 composite starting from 600 cm^−1^.

The characteristic absorption of the CPC functional group was mainly located at 560–600 cm^−1^, 1020–1120 cm^−1^, and 1650 cm^−1^. The typical vibration bands of PO_4_^3−^ in the HA structure were observed at 560–600 cm^−1^ and 1020–1120 cm^−1^, and the stretching band of OH^−^ was observed at 1650 cm^−^^1^ (Figure 4a). For the C/0.25 composite, the absorption band at 875–880 cm^−1^ indicated the presence of HPO_4_^2−^, but no obvious characteristic band of OH^−^ was observed at 1650 cm^−1^. From the above XRD results, it can be seen that there was an interaction between the polar functional groups (such as carboxyl, carbonyl, and amino) in the hydrogel and the inorganic phase (DCPA, TTCP, and apatite) to control the nucleation of apatite, thereby changing the preferred orientation in the orderly growing crystal. Therefore, the FTIR spectrum of the C/0.25 composite was different from that of the CPC-only. A study reported that the effect of the combination of polysaccharide or gelatin in CPC on the crystallinity of CPC was minimal [37]. However, another study revealed that gelatin accelerated hydraulic reactions of CPC paste, in which reactants were immediately converted into nanostructured apatite precipitates after hardening [35]. Gelatin molecules induced 4–10% macropores (10–300 μm) in the cement structure and decreased the initial setting time by approximately 190%. The composition analysis using X-ray diffraction (XRD) indicated that the final product phase was mainly related to the bone cement that was used. In the present study, the addition of hydrogel beads slightly delayed the phase transition of CPC. After 7 days of soaking, the composition of the raw materials could still be observed; however, the final product phase was still converted into apatite; this result is identical to that obtained through a single CPC reaction.

### 3.2. In Vitro Interaction of Osteoprogenitor D1 Cells and Materials

#### 3.2.1. Short-Term Cell Attachment of CPC-Only and C/0.25 Composite

**Figure 5:**
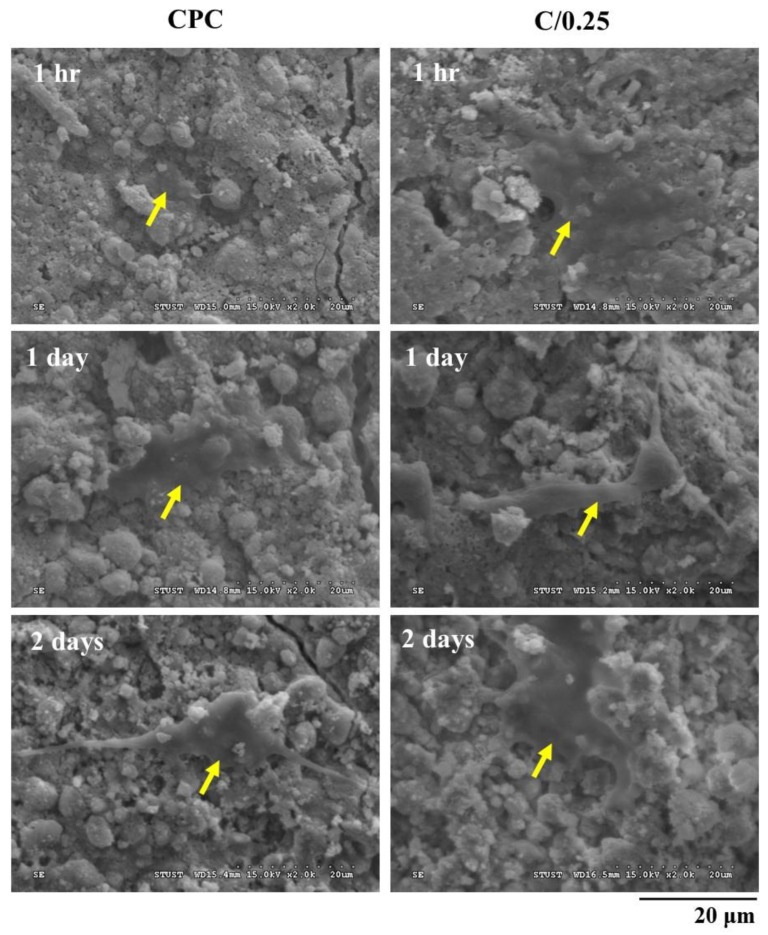
Short-term cell attachment of CPC-only and C/0.25 composite co-cultured with osteoprogenitor D1 cells (arrows indicate D1 cells on the surface).

Osteogenic progenitor cells, a bone marrow mesenchymal stem cell (MSC) line cloned from Balb/C mice, namely D1 cells, were cultured on the surface of CPC for 1 h, and spherical precursor osteoblasts were observed on the surface of the CPC. After 1 day of culture, the cell nucleus flattened on the CPC plane, indicating suitable adhesion; after 2 days of culture, the filopodia of D1 cells were formed and new adhesion sites were created (Figure 5). After D1 cells were incubated in the C/0.25 composite for 1 h, flat cells were observed on the surface of the C/0.25. After 1 day of culture, the osteoprogenitor D1 cells exhibited obvious filopodia, indicating that the C/0.25 composite had favorable cell affinity. As gelatin is composed of amino acids and comprises numerous glycine, proline, and 4-hydroxy proline residues, it is highly suitable for cell attachment and proliferation [31,32].

#### 3.2.2. Long-Term Cell Proliferation and ALP Activity

**Figure 6:**
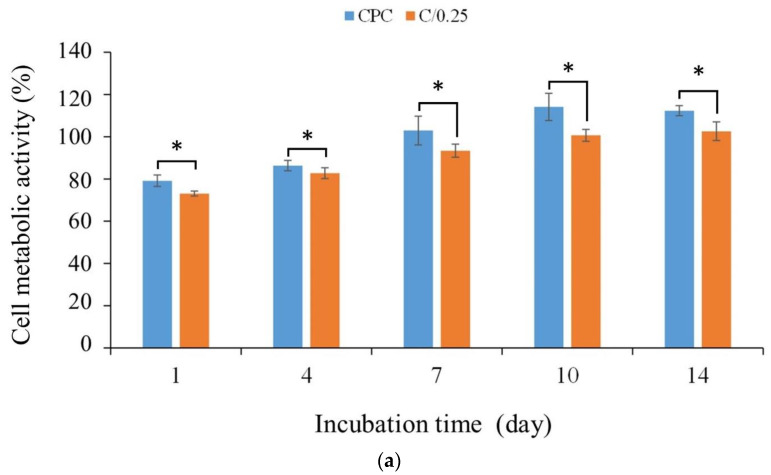

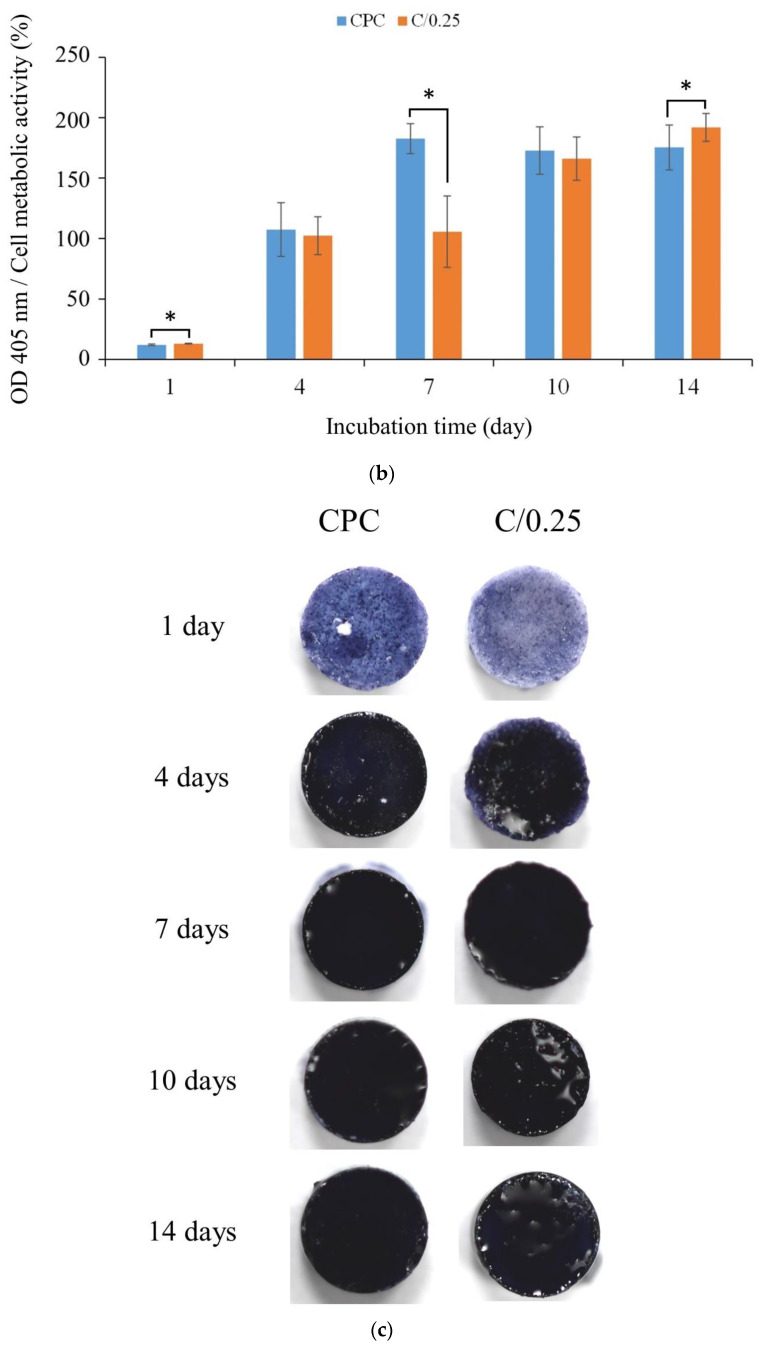
(**a**) Cell proliferation, (**b**) D1 cell ALP activity, and (**c**) ALP qualitative staining of CPC-only and C/0.25 composite co-cultured with osteoprogenitor D1 cells (*n* = 3, * *p* < 0.05).

Figure 6a shows the quantitative results of the long-term proliferation of D1 cells in CPC-only and C/0.25 composite materials. The results showed that with the extension of the culture time, cell proliferation led to an increase in cell metabolic activity. When D1 cells were cultured in CPC-only and C/0.25 composite after 7 days as an early sign, the cell metabolic activity gradually reached a plateau (Figure 6b). Compared with the control group, the cell metabolic activity of each group exceeded 70%, indicating that it did not cause cytotoxicity. Only the cell metabolic activity of the CPC-only and C/0.25 composite did not increase significantly on the 7th day. In the CPC-only and C/0.25 composite, the cell metabolic activity continued to increase until day 10, plateaued on day 14, and did not exhibit a downward trend. Despite the trend being the same, the cell metabolic activity of the CPC-only was higher than that of the C/0.25 composite at all culture time points.

The ability to promote the ALP secretion of osteoprogenitor D1 cells gradually increased from days 1 to 7 when CPC-only was cultured alone and then stabilized (Figure 6b). For the C/0.25 composite, its tendency to promote ALP could be divided into two stages due to hydrogel incorporation. The first stage was from days 1 to 4, and the second stage was from days 7 to 10. Comparison of the CPC-only and C/0.25 composite groups revealed that the CPC-only was significantly more effective than the C/0.25 d at promoting ALP secretion on days 4 and 7 of culture (Table 1). On the 10th and 14th days, the ability of the C/0.25 composite to promote the ALP secretion was not significantly different from that of D1 cultured with CPC-only (Figure 6b). ALP staining is often used to further confirm the results of quantitative analysis of osteoprogenitor D1 ALP activity. Deeper staining indicates that more ALP is secreted and the CPC exhibited deeper staining than the C/0.25 composite at days 1 and 4 of culture (Figure 6). When the culture time increased, the staining of the two groups was similar, and the results were consistent with the trend presented in Figure 6b.

For cell proliferation and ALP measurement, the addition of hydrogel beads to CPC bone paste did not increase the proliferation of osteoprogenitor D1 cells but affected subsequent ALP performance. A study reported that the addition of more gelatin to CPC can effectively promote osteoblastic responses that enhance nanoapatite precipitation [38]. Therefore, the in situ deposition of nanoapatite on the hydrogel might play a key role in biological activity [39]. The hydrogel exposed from the CPC/0.25 composite would take time to release from the CPC matrix. Then, it can mimic the extracellular matrix, provide a microenvironment for cell proliferation and differentiation, and further induce tissue regeneration. The delay time shown by the C/0.25 composite indicates that the second stage of D1 cell culture increased significantly from day 7 to day 10 (Table 1). Although the CPC used in the present study has the same composition as the CPC used in other studies, differences still exist in terms of the molar ratio and surface treatment [15,35]. Our previous research showed that CPC-only developed in-house has the powerful ability to enhance cell attachment and osteogenic differentiation; therefore, the C/0.25 composite cannot positively affect in vitro proliferation and osteogenic function [29]. Furthermore, in the present study, 2–3 mm gelatin particles were directly added to the CPC matrix to provide an exposed template for creating submeter pores for cells (and not to verify the nucleation sites of nanoapatite), and this may be the cause of the difference in D1 cell culture performance [40].

### 3.3. Histology of CPC, Hydrogel Beads, and C/0.25 Composite In Vivo

**Figure 7:**
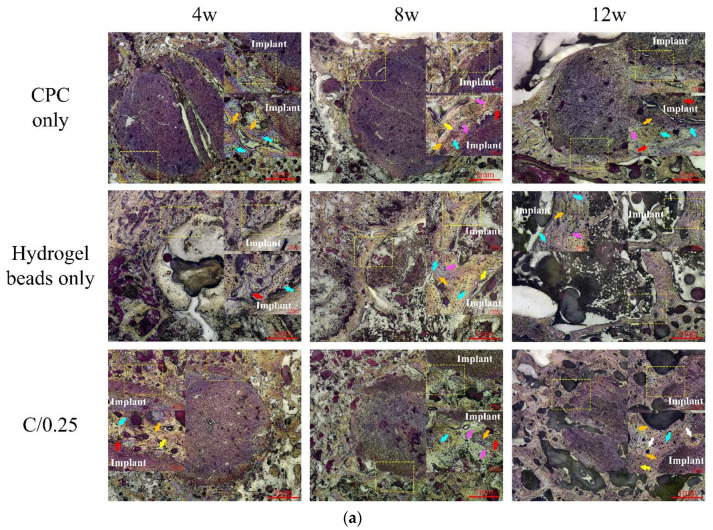

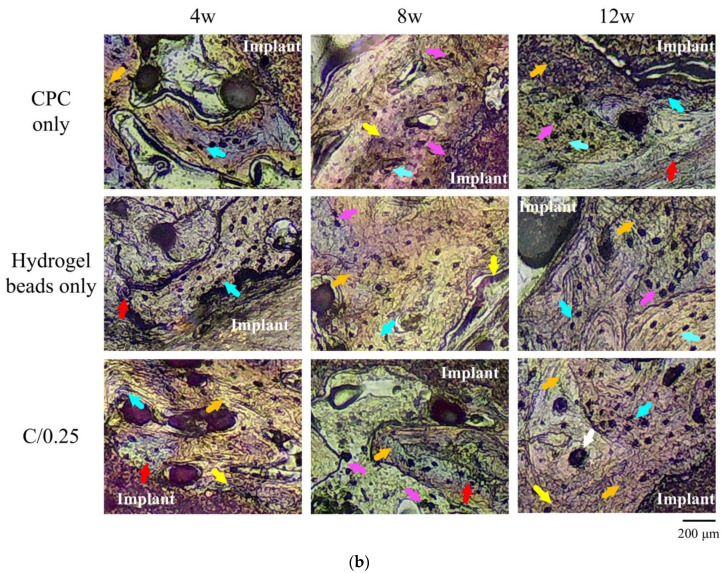
Histological observation (**a**) and partial enlarged image (**b**) of CPC-only and C/0.25 composite after 4, 8, and 12 weeks of implantation. Red arrow: blood vessels; yellow arrow: osteocyte; orange arrow: trabecular bone; blue arrow: osteoblasts; pink arrow: osteoclasts; white arrow: haversian canal.

Figure 7a presents the observation results of cylindrical implants in the intramedullary osseointegration model of the distal femur of rabbits at 4, 8, and 12 weeks. Compared with the two groups of CPC-only and C/0.25 composite, the implant using hydrogel beads-only exhibited a clear interface between the implant and bone tissue at 4 weeks after implantation. The cavity indicates that there was no obvious bone formation after hydrogel-only implantation (Figure 7b). This may be due to the rapid degradation of implants that use only hydrogel. In the CPC-only and C/0.25 composite groups, bone tissue was well integrated with the implant, and no obvious gap or separation was observed. After 4 weeks of implantation, no significant absorption was noted in the CPC-only and C/0.25 composite groups.

Eight weeks after the hydrogel beads were implanted, a clear interface was obvious, and a gap was present between the implant and surrounding bone, which was verified to be soft tissue (Figure 7b). In the CPC-only and C/0.25 composite, no gap was observed between the implant and surrounding bone. In addition, numerous neatly arranged osteoblasts were observed, indicating that new bone was forming and bone regeneration was active. The reduction in the planting area of the C/0.25 composite was greater than that of the CPC-only. By combining the hydrogel beads into the CPC matrix, the absorption of the implant material was promoted, which may explain why fewer residual implants were observed.

**Figure 8:**
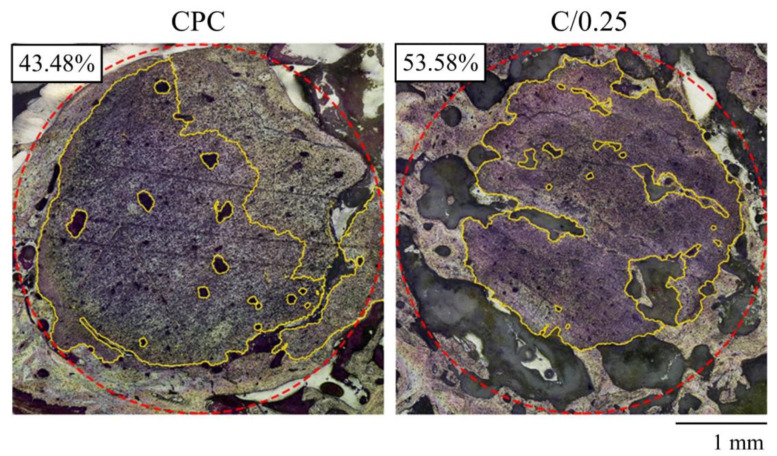
A quantified absorption rate of CPC-only and C/0.25 composite after 12 weeks of implantation (red dotted line indicates an original bone defect, which is a circular implant with a diameter of 4 mm; yellow range indicates the area of the residual implant, and the number indicates absorption).

As we mentioned, we intended to use the minimum number of animals to study the natural biological interactions. As the sample preparation for decalcification is an extremely precise and irreversible process, it is unfortunate that no effective statistical results for calculating the bioabsorption of the implant could be obtained after 12 weeks of implantation. In Figure 8, the decalcified images provide certain evidence that numerous new trabecular bones formed at the junction of the CPC-only and C/0.25 composite, and the calculated implant absorption rate of the C/0.25 composite was higher than that of the CPC-only, indicating, at least in the case comparison, that there were more new bones present in the C/0.25 composite. The absorption rates of the CPC-only and C/0.25 composite were 43.48 vol.% and 53.58 vol.%, respectively. According to past findings [34], the formation of holes after hydrogel beads degrade is beneficial to angiogenesis, which, in turn, promotes bone formation.

In our previous research [12,41], we found that the absorption rate of CPC-only after injection is not conducive to destroying the integrity of the CPC in vivo, because the absorption is less than 50 vol.%. For example, CPC-only has an average of 44.9 vol.% and a standard deviation of 4.2% in the absorption value 12 weeks after implantation [36]. It is worth noting that in this study, the absorption rate of the C/0.25 composite was greater than 50%. The results showed that adding hydrogel beads in CPC may potentially increase the absorption rate after implantation. In one study, gelatin was added to CPC powder and implanted in the trabecular defect of the distal femur [42]; with the addition of 10% gelatin powder to the CPC, after 12 weeks of implantation, the residual rate of the implanted compound was 45.8%. In the present study, the residual rate of the C/0.25 composite was 46.42%, which is similar to the aforementioned result. Another study also verified through pig tibia implantation that after CPC was mixed with 10% gelatin (by weight) and chondroitin sulfate, the absorption rate of the implant material was 85% after 8 weeks of implantation [34]. The results verified that through the degradation of gelatin particles, interconnected macropores can form inside a CPC matrix, leading to cell infiltration, biological resorption of a specimen, and subsequent formation of new bone. The aforementioned results indicate that the findings reported in the literature are comparable to those reported in the present study; however, the volume percentage of hydrogel beads added in the present study was 25%. Compared with the addition of 10 wt.% gelatin particles in a CPC matrix, a composite comprising CPC and hydrogel beads can improve the osteointegration and implant absorption rate of an implant in the long term.

The present study discovered that different implant materials exhibit distinct absorption rates and bone formation patterns in vivo. As CPC may be relatively dense, no pores are present after a hardening reaction, and the absorption mode of CPC involves gradual absorption from the outer edge of an implant. Therefore, most new bone is formed along the periphery of the implant. Furthermore, when the implant material is a granular powder, the formation of new bone along the edges of particles often causes the local implant powder to collapse or aggregate, resulting in relatively slow absorption. The appearance of the bone particles used in clinical repair is similar. A bone scaffold is deposited at the bottom of a bone defect, and the scaffold is not easily absorbed for bone tissue generation, which affects bone repair. This study proposes that adding hydrogel beads to CPC can enhance the feasibility of bone tissue repair. As hydrogel beads are biodegradable polymers, they can absorb a large amount of liquid such that they become rich in blood and platelet-derived growth factors during implantation, which promotes the growth of blood vessels into the implant sites to supply nutrients and increase metabolism. After the hydrogel beads are degraded, the original dense and nonporous CPC matrix becomes porous, which is conducive to absorption and promotes bone formation.

## 4. Conclusions

With consideration of the limitations of the biomechanisms between implants and biological reactions, the results indicate that 25%-volume biodegradable hydrogel beads can mix with quick-setting CPC. This newly developed composite is moldable and has high resistance to disintegration after contact with fluids. The overall mixing process for the C/0.25 composite can be completed within 5 min, making the composite suitable for clinical application. The composite does not affect phase change or cause cytotoxicity. Although it cannot improve the proliferation of osteoprogenitor cells, it is equally competitive in the subsequent ALP activity against that of the CPC-only in vitro. The application potential of adding hydrogel beads is to increase the absorption rate of CPC in vivo. In the future, this hydrogel/CPC composite, which was developed for clinical applications, can be used to address the problems associated with the compact and nonporous structure after CPC implantation, which is not osteoconductive to a dental filling.

## Figures and Tables

**Table 1:** Results of the Tukey’s honestly significant difference (HSD) post hoc in Figure 6a,b for the comparisons of cell metabolic activity and ALP, with groups that include the same letter denoting statistically nonsignificant differences and * representsa significantly difference.

Two-Way ANOVA	Source	DF	Sum of Squares	Mean Square	F Ratio	
Cell Metabolic Activity	Model	9	1.5989	0.1777	116.1440	
	Error	80	0.1224	0.0015	Prob. > F	
	C. Total	89	1.7212		<0.0001 *	
	groups	1	0.1621	106.0013	<0.0001 *	
	days[groups]	8	1.4368	117.4119	<0.0001 *	
Group comparisons (Levels not connected by the same letter are significantly different)						
[CPC]10d	A					
[CPC]14d	A					
[CPC]7d		B				
[C/0.25]14d		B				
[C/0.25]10d		B				
[C/0.25]7d			C			
[CPC]4d				D		
[C/0.25]4d				D	E	
[CPC]1d					E	
[C/0.25]1d						F
Groups are significantly different at *p* < 0.05.						
**Two-Way ANOVA**	**Source**	**DF**	**Sum of Squares**	**Mean Square**	**F Ratio**	
ALP	Model	9	36.6062	4.0674	141.8610	
	Error	77	2.2077	0.0287	Prob. > F	
	C. Total	86	38.8139	-	<0.0001 *	
	groups	1	0.4335	15.1193	0.0002 *	
	days[groups]	8	36.0612	157.2173	<0.0001 *	
Group comparisons (Levels not connected by the same letter are significantly different)						
[C/0.25]14d		A				
[CPC]7d		A				
[CPC]14d		A				
[CPC]10d		A				
[C/0.25]10d		A				
[CPC]4d				B		
[C/0.25]7d				B		
[C/0.25]4d				B		
[C/0.25]1d					C	
[CPC]1d					C	
Groups are significantly different at *p* < 0.05.						

## Data Availability

Not applicable.
